# Molecular Modeling Analysis Provides Genotype–Phenotype Correlation Insights in a Patient with Ankyloblepharon-Ectodermal Dysplasia-Clefting Syndrome

**DOI:** 10.3390/genes14061246

**Published:** 2023-06-10

**Authors:** Anna Douka, Lambros Goutzanis, Dimitrios Vlachakis, George P. Chrousos, Christos Yapijakis

**Affiliations:** 1 Unit of Orofacial Genetics, 1st Department of Pediatrics, School of Medicine, National Kapodistrian University of Athens, “Aghia Sophia” Children’s Hospital, 11527 Athens, Greece; douka.anna1998@gmail.com; 2 Laboratory of Molecular Genetics, Cephalogenetics Center, 17675 Athens, Greece; 3 Department of Oral and Maxillofacial Surgery, School of Dentistry, National Kapodistrian University of Athens, 11527 Athens, Greece; lgoutzan@yahoo.gr; 4 Laboratory of Genetics, Department of Biotechnology, School of Applied Biology and Biotechnology, Agricultural University of Athens, 11855 Athens, Greece; dimvl@aua.gr; 5 University Research Institute for the Study of Genetic and Malignant Disorders in Childhood, Choremion Laboratory, “Aghia Sophia” Children’s Hospital, 11527 Athens, Greece; chrousge@med.uoa.gr

**Keywords:** ectodermal dysplasia, ankyloblepharon, AEC syndrome, *TP63* gene, case report, p63, protein structure, sterile α motif domain

## Abstract

Ankyloblepharon-ectodermal defects-cleft lip/palate (AEC) syndrome is a rare autosomal dominant disorder. AEC is caused by mutations in the *TP63* gene that encodes the tumor suppressor p63 protein, itself involved in the regulation of epidermal proliferation, development, and differentiation. We present here a typical AEC case of a four-year-old girl with extensive skin erosions and erythroderma of the scalp and the trunk, and to a lesser extent of the limbs, nail dystrophy on the fingers and toes, xerophthalmia, a high-arched palate, oligodontia, and hypohidrosis. Mutation analysis of the *TP63* gene detected a de novo missense mutation in exon 14 (c.1799G>T; p.Gly600Val). We discuss the phenotype–genotype correlation by presenting the clinical features of AEC in the patient, and the effect of the detected mutation in p63 structure and function using protein structural modeling, in view of similar cases in the literature. We performed a molecular modeling study in order to link the effect on the protein structure level of the missense mutation G600V. We noted that the introduction of the bulkier Valine residue in place of the slim Glycine residue caused a significantly altered 3D conformational arrangement of that protein region, pushing away the adjacent antiparallel α helix. We propose that the introduced locally altered structure of the G600V mutant p63 has a significant functional effect on specific protein–protein interactions, thus affecting the clinical phenotype.

## 1. Introduction

Ankyloblepharon-ectodermal dysplasia-cleft lip/palate (AEC) syndrome (OMIM 106260), also known as Hay–Wells syndrome, was originally identified in 1976 [1]. The eyes, hair, skin, nails, dentition, and lip and/or palate are the primary structures impacted [1,2,3]. AEC is an autosomal dominant condition with an unknown prevalence, which belongs to a large heterogenous group of ectodermal dysplasias (EDs) that range between 1–70/100,000 [2,3,4]. Typically, EDs include embryonic ectodermal abnormalities, which can affect the skin, hair, nails, teeth, sweat glands, lips, palate, hands, and feet [4,5,6].

Ankyloblepharon-ectodermal dysplasia-cleft lip/palate (AEC), Ectrodactyly-ectodermal dysplasia clefting (EEC), Rapp–Hodgkin (RHS), ADULT (Acro-dermato-ungual-lacrimal-tooth), and Limb-mammary (LMS) are some of the ED syndromes that are brought on by *TP63* gene mutations [7,8]. This gene, which is a member of the *TP53* family, is essential for the growth and maintenance of stratified epithelium tissues, such as the skin; it also plays a key role in quality control by keeping track of the genetic integrity of oocytes.

Three different types of genetic variations in the *TP63* gene are responsible for AEC syndrome: point mutations in the region encoding the sterile α motif (SAM) domain, point mutations in the transactivation-inhibitory (TI) domain, and frameshift mutations that are C-terminal to the oligomerization domain [9,10]. According to the Human Gene Mutation Database (HGMD Professional 2021.4, hgmd.cf.ac.uk, accessed on 28 April 2023)), AEC syndrome has been associated with 31 mutations in the *TP63* gene, with most of them being missense/nonsense in the SAM domain. The SAM domain of p63 may be implicated in protein–protein interactions with over 40 other proteins that are involved in transcriptional regulation and ectodermal development [9]. Mutations located in the SAM domain of p63 result in the AEC syndrome by eliminating the contact with ABBP1 (apobec-1-binding protein-1), and by modifying the FGFR-2 pathway, which results in abnormal differentiation [11]. All known *TP63* gene mutations causing AEC syndrome are related to the obstruction of normal keratinocyte proliferation, differentiation, and survival, and result in the observed skin abnormalities of the disorder [12].

Here, we present a patient with AEC syndrome caused by a novel point mutation in the SAM domain of the *TP63* gene. Using protein structural modeling, we indicate the disruption of the protein structure and function that the observed mutation causes. In addition, we briefly but critically review the published work on the syndrome.

## 2. Materials and Methods

### 2.1. Data Collection

The study involved two individuals from a European family that included an ectodermal dysplasia patient, and a healthy parent. Clinical examination established the diagnosis of ectodermal dysplasia in the patient and the absence of the disorder in the healthy individual. After genetic counseling, the parent signed an informed consent form before subsequent blood sampling for genomic DNA isolation and genetic testing. The Bioethics Committee of the University Research Institute for the Study of Genetic and Malignant Disorders in Childhood at the School of Medicine of the National Kapodistrian University of Athens ethically approved the study (RPURI9002).

### 2.2. Sequence Analysis

After informed consent, genomic DNA was isolated from the white blood cells of the patient and her healthy mother for molecular genetic analysis in order to determine the exact ectodermal dysplasia subtype. Total DNA isolation was performed using Nucleospin^®^ Blood Quickpure kit (Macherey Nagel GmbH, Düren, Germany), following the guidelines of the manufacturer. Subsequently, whole exome sequencing (WES) was performed with the patient and mother’s DNA, for identification of the mutation using Novaseq 6000 (Illumina, San Diego, CA, USA). For confirmation, targeted DNA sequencing of the *TP63* gene region containing the mutation was carried out for both the patient and her healthy parent, using an automated capillary sequencer ABI 3730 XL Analyzer (Applied Biosystems, Waltham, MA, USA). The sequence of used primers is as follows: 5′-GTGGATCAATAGATTCAGATCA-3′ (Forward) and 5′-GGTACAGTTTCAACTTCAGTAAG-3′ (Reverse). The resulting PCR product of the *TP63* gene region containing the mutation is 369 bp.

### 2.3. Data, Sequence Alignment, and Template

3D coordinates were obtained from the X-ray solved crystal structure of the p63a SAM domain mutant involved in the AEC syndrome, using RCSB code: 2Y9T. The full amino acid sequence for human p63 was obtained from the GenBank database (Accession number: NP_003713.3). Using the Gapped-BLAST through NCBI, the 2Y9T homologous crystalized protein was identified and was used as a template for the mutation modeling of the p63 structure. The online version of ClustalW was used for sequence alignment. Hidden Markov models were used to repeat the alignment, and produced the same result as that obtained using ClustalW, since there are numerous anchoring conserved motifs throughout the alignment.

### 2.4. Energy Minimization and Molecular Dynamics

The removal of the residual geometrical strain in each molecular system was performed via minimizations of energy using the Amber99 force field, as implemented into the Gromacs suite, version 4.5.5. All Gromacs-related simulations were made through our graphical interface, which we developed previously. At this stage, an implicit generalized born (GB) solvation was chosen in an attempt to accelerate the process of energy minimization. Subsequently, molecular systems were subjected to unrestrained molecular dynamics simulations (MDS) using the Gromacs suite, version 4.5.5. MDS took place in an SPC water-solvated, periodic environment. Water molecules were added using the truncated octahedron box extending 7 Å from each atom. Molecular systems were neutralized with counter-ions, as required. For this study, all MDS were performed using an NVT ensemble in a canonical environment, at 300 K, and a step size equal to 2 femtoseconds for a total of 100 nanoseconds of simulation time. An NVT ensemble requires that the number of atoms, volume, and temperature remain constant throughout the simulation.

### 2.5. Mutant Model Evaluation

The model quality and reliability in terms of its 3D structural conformation were evaluated as important issues for the viability of this study. The produced models were initially evaluated within the Gromacs package using a residue packing quality function, which depends on the number of buried non-polar side chain groups and on hydrogen bonding. Furthermore, the suite PROCHECK was used for additional evaluation of the quality of the produced models. Lastly, the molecular operating environment (MOE) suite was employed in order to evaluate the 3D geometry of the models in terms of their Ramachandran plots, omega torsion profiles, phi/psi angles, planarity, C-β torsion angles, and rotamer strain energy profiles.

## 3. Results

### 3.1. Case Report

**Figure 1:**
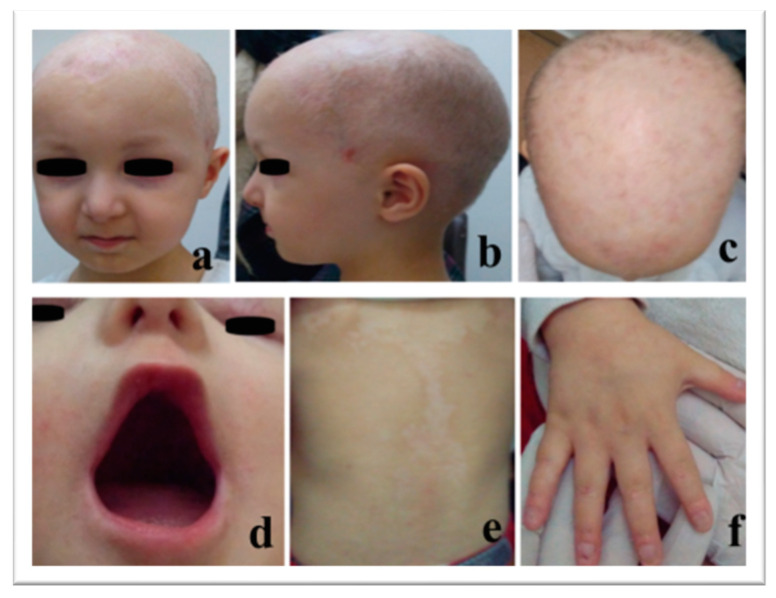
Clinical appearance of the patient; (**a**–**c**): scalp with extensive skin erosions; (**d**): oligodontia and high-arched palate with no clefting; (**e**): trunk with areas of darkened or faded skin color; (**f**): dystrophic nails.

A four-year-old female with no family history of ectodermal dysplasia was clinically diagnosed with AEC syndrome. She was the first child of healthy parents with a family history free from similar cases; her father is from Italy and her mother from Greece. Her male brother is normal. Physical examination of the patient revealed extensive skin erosions and erythroderma of the scalp and the trunk, and to a lesser extent of the limbs, nail dystrophy on the fingers and toes, xerophthalmia due to lack of tears, sparse eyelashes and eyebrows, a high-arched palate (but no sign of clefting), oligodontia (missing 11 primary teeth), areas of darkened or faded skin color, and hypohidrosis. The combination of her oligodontia and her parents speaking different languages perhaps caused her to have a speech delay for her age. Due to lack of tears, she had to use eye drops for hydration, at least every three hours, depending on the weather. As a result, her corneal membrane was very sensitive and had suffered frequent damage. She has photophobia, which has forced her to wear sunglasses every time she is exposed to the sun. in the skin of her head also shows increased sensitivity to light, with healing difficulties (Figure 1).

### 3.2. Molecular Genetic Analysis

**Figure 2:**
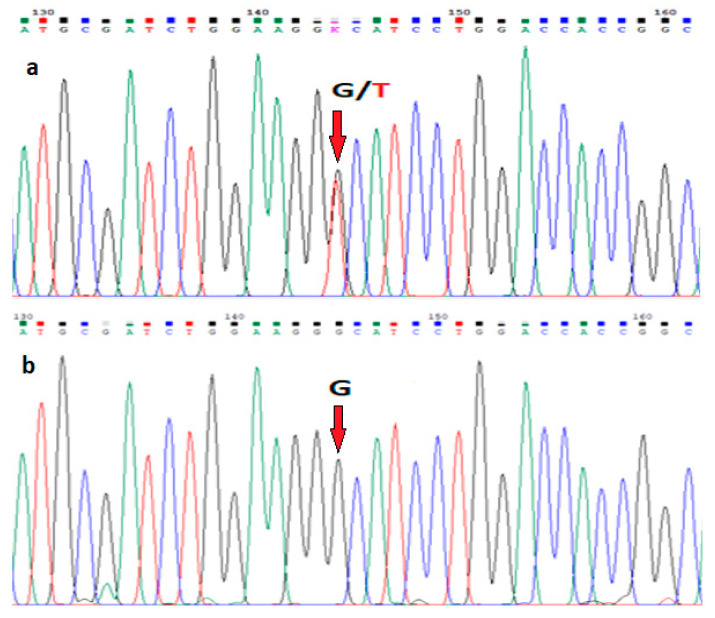
DNA sequencing findings of exon 14 of the *TP63* gene in (**a**) the patient who is heterozygous for mutation c.1799G>T (p.Gly600Val), and (**b**) the mother who is a homozygous for the normal 1799G allele.

**Figure 3:**
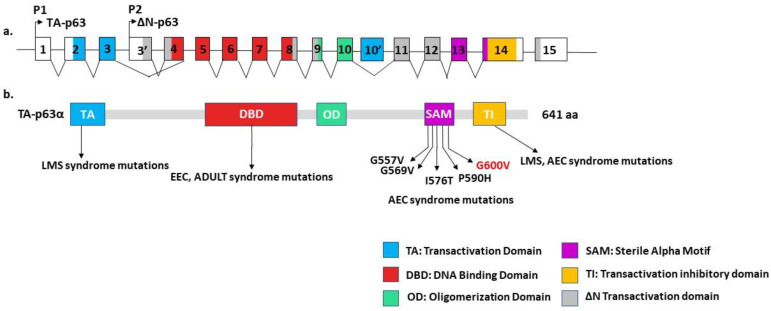
(**a**) Schematic representation of intron/exon structure of the human p63 gene. Two promoters (P1 and P2) give rise to two isoforms at the N-terminus, the transactivating (TA) and N-terminally truncated (ΔN) isoforms. Exons are colored according to the functional domains, as indicated. (**b**) Schematic representation of the human p63 protein TA-p63α isoform of 641 aminoacids (aa). Various mutations in the human *p63* gene cause different syndromes (AEC: Ankyloblepharon-ectodermal dysplasia-cleft lip/palate; EEC: Ectrodactyly ectodermal dysplasia clefting; ADULT: Acro-dermato-ungual-lacrimal-tooth; LMS: Limb-mammary syndrome), as indicated in the transactivation domain (TA), DNA-binding domain (DBD), oligomerization domain (OD), sterile α motif (SAM), and transactivation inhibitory domain (TI). The SAM domain contains most of the missense mutations that cause AEC syndrome, and the mutation G600V reported here.

A peripheral blood DNA sequence analysis was performed to confirm the putative clinical diagnosis. Sequencing of the patient’s *TP63* gene detected a heterozygous missense mutation c.1799G>T in exon 14 of the sterile α motif (SAM), causing an amino acid substitution p.Gly600Val (Figure 2). The observed mutation is pathogenic, as it has been previously detected in another AEC patient [9,13]. DNA sequencing analysis of the mother did not reveal any mutations in the *TP63* gene. Therefore, the diagnosis of ankyloblepharon-ectodermal defects-cleft lip/palate (AEC) syndrome was confirmed in the patient, as the disorder is apparently caused by a de novo dominant mutation in the SAM domain of the *TP63* gene, in which most AEC-causing mutations reside (Figure 3).

### 3.3. Protein Structural Modeling

**Figure 4:**
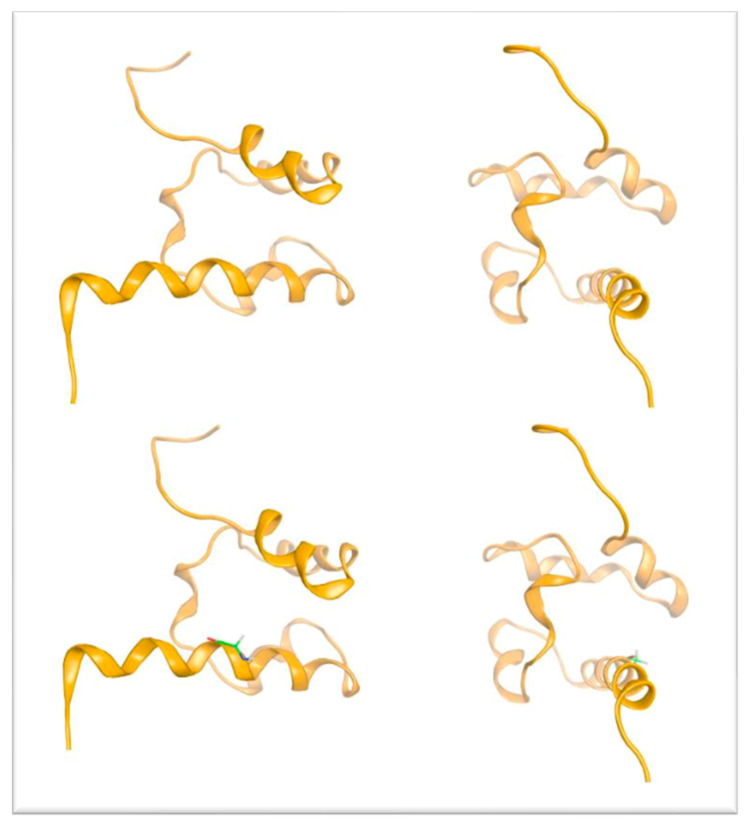
3D structure of the human p63 (orange ribbon representation). The green Gly600 residue is shown by the Corey–Pauling–Koltun stick representation on the lower plane.

**Figure 5:**
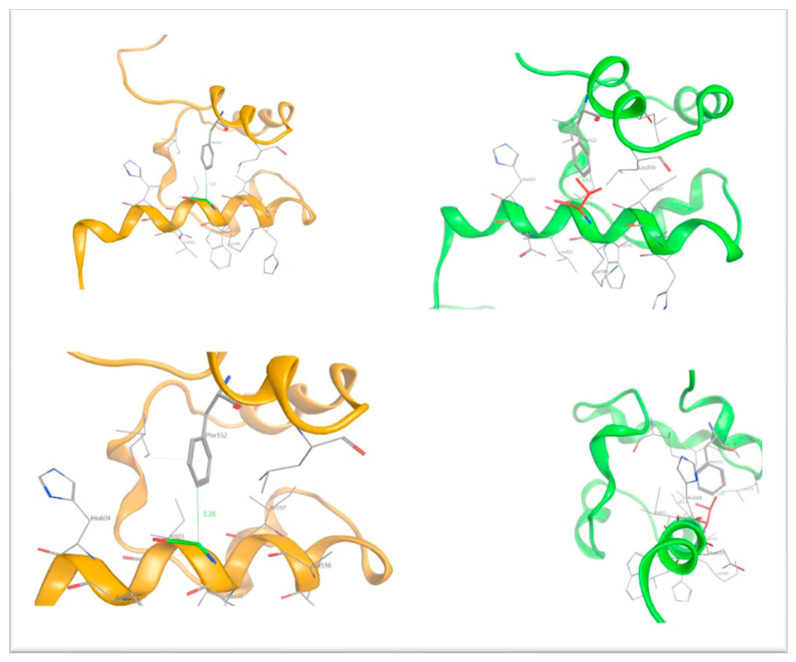
3D structural representation of the wild-type p63 (orange ribbon representation) and the mutant G600V mutant (green ribbon representation). The distances between the Gly600 and Phe552 as well as Val600 and Phe552 are shown in Angstroms.

**Figure 6:**
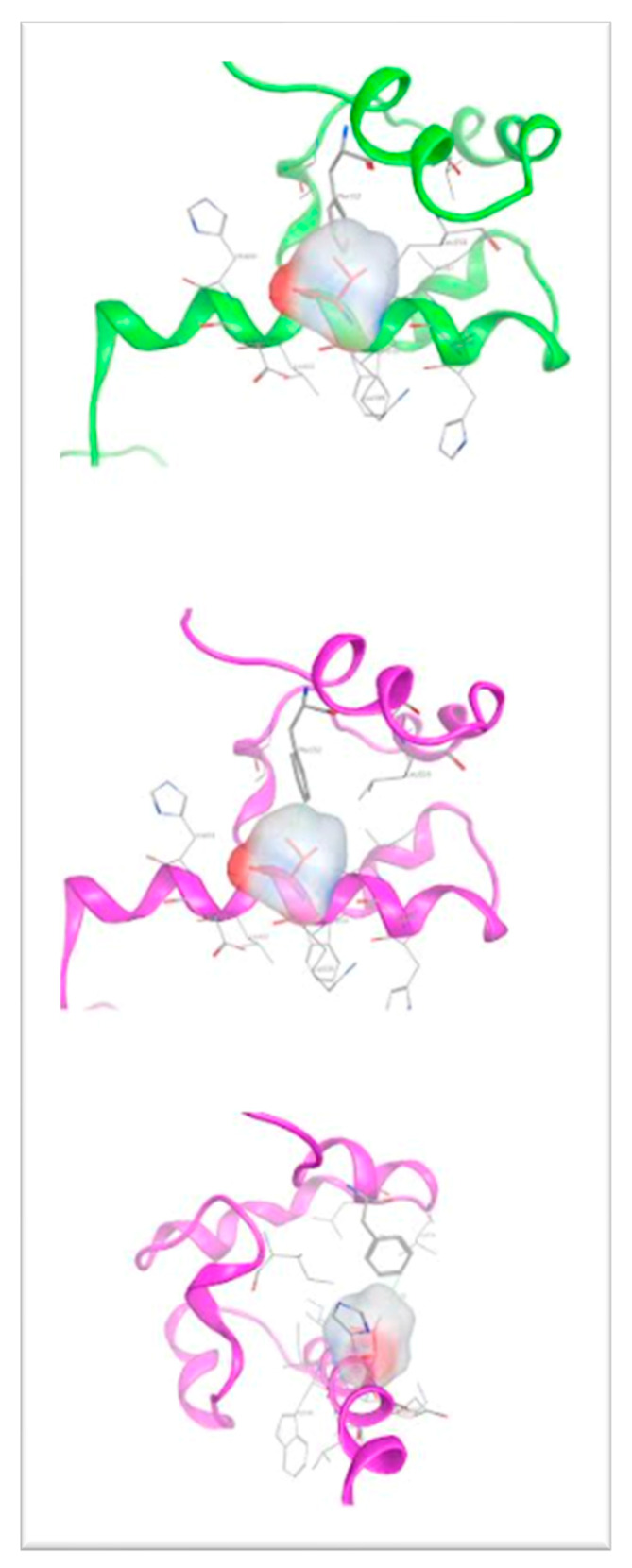
3D model of the mutant Gly600Val p63 prior to and upon molecular dynamics simulation (green and magenta ribbon representation, respectively).

To shed light on the underlying molecular mechanism of the detected point mutation, a protein modeling study was performed. We attempted to link the effect of the introduced mutation to the phenotype by focusing on the molecular level of the protein structure. The introduced substitution of a Valine residue in position 600 of the polypeptide chain of p63 was found to cause significant structural changes. The main reason is that the introduced Valine residue is bulkier than the native Glycine residue at this position. Figure 4 is the molecular representation of the wild-type p63, highlighting the position of Gly600 in the middle of an α helix. Superimposing the wild-type p63 (Gly600) onto the modeled mutant (Gly600Val) p63 revealed that the bulkier Valine does not have enough conformational space to accommodate its longer side chain. As a result, the introduced Valine residues clashes with the nearby Phe552 residue, which is located in an adjacent antiparallel α helix (Figure 5). The distance of the Gly600 residue from the neighboring Phe552 residue was calculated to be 3.25 Angstroms in the wild-type p63 (orange ribbon representation, Figure 5), while in the mutant model, the distance between the introduced Val600 and the Phe552 was only 0.63 Angstroms (green ribbon representation, Figure 6).

**Figure 7:**
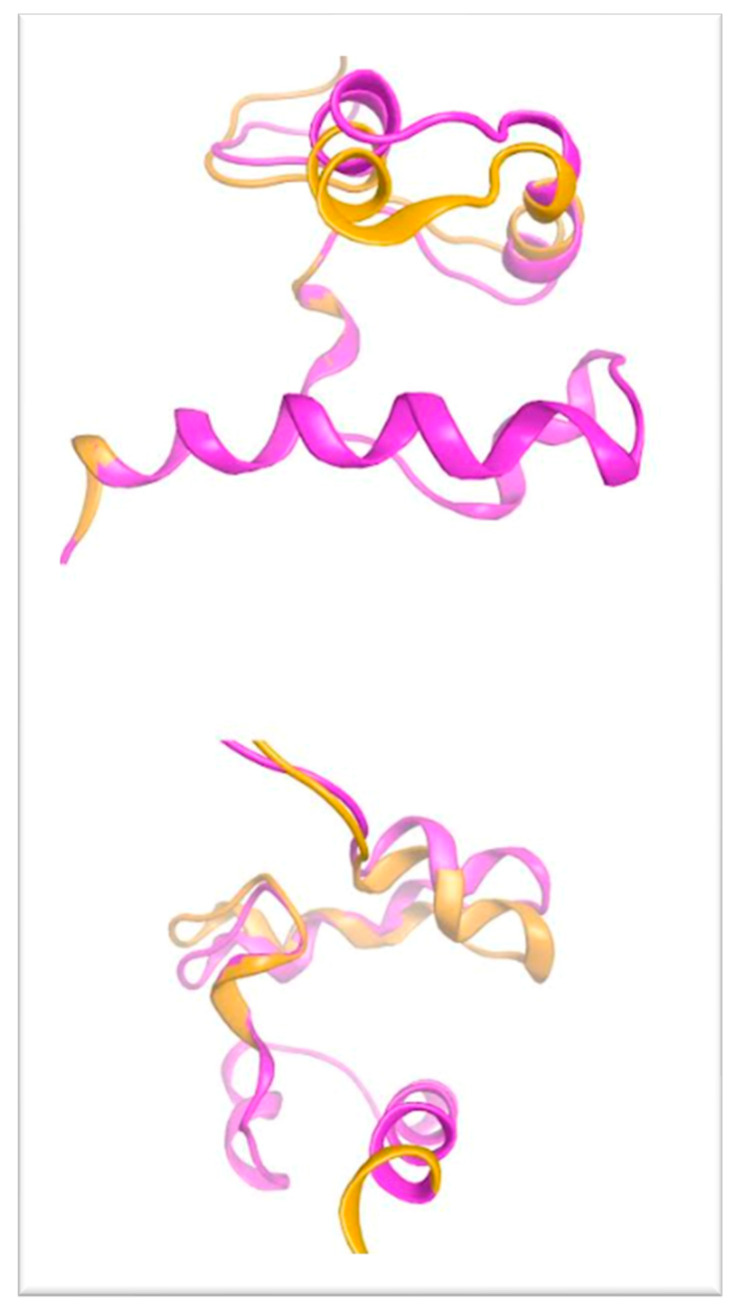
Superposition of the wild-type p63 (orange ribbon) with the final structure of the Gly600Val mutant of p63 (magenta ribbon representation) upon molecular dynamics simulation. Note the significant conformational change introduced to the structure of p63 by the Gly600Val mutation.

In an effort to relax the modeled structure of the G600V mutant p63 and reduce its increased thermodynamic energy (due to the clashing residues), the above-mentioned model was subjected to energy minimization followed by a molecular dynamics (MD) simulation. We found that the final structure of the G600V mutant model of p63 has significant structural differences upon the MD simulation, when compared to the structure prior to the simulation (the wild-type secondary and tertiary structure). The distance between Val600 and the Phe552, which was only 0.63 Angstroms in the original model, increased to 3.16 Angstroms. This was because the introduced balkier Valine required more space for its side chain. As a result, the adjacent antiparallel α helix bearing the Phe522 residue was pushed away, thus significantly altering the 3D conformational arrangement of the G600V mutant model p63 (magenta ribbon representation, Figure 6). The outcome of the structural effect of the introduced G600V mutation on the human p63 is visualized in the superimposed pre- and upon molecular dynamic models of p63 (Figure 7). We propose that the structural alteration introduced to the G600V mutant p63 has a significant functional effect on this protein, thus affecting the clinical phenotype.

**Figure 8:**
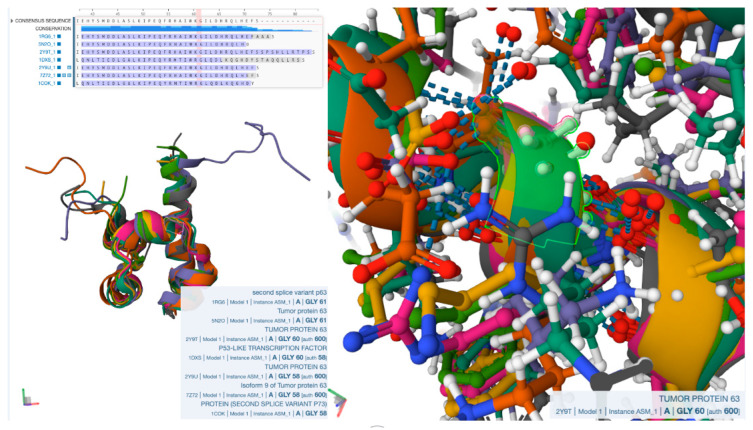
Sequence alignment and structural superposition of 7 p63 proteins sharing more that 50% sequence identity with the Protein Data Bank entry 2Y9T used in this study. **Left**: (**Top**) Sequence alignment in the proximity of the 600 position, validating that Gly is conserved. (**Bottom**) Structural superposition of the seven p63 homologues, confirming that p63 structure is conserved. **Right**: Zoomed-in illustration of Gly600 position, revealing that the latter residue has a position that is spatially conserved in all superposed structures.

It is well known that the p63 protein plays important functional roles in regulating cell proliferation and differentiation during embryonic development, as well as in the maintenance of several tissues, including the skin. Overall, functional models of p63 suggest that its loss of function can lead to a variety of pathological conditions, including cancer. In an effort to address all of the above from a structural modelling point of view, we searched the Research Collaboratory for Structural Bioinformatics (RCSB) Protein Data Bank for protein homologues sharing at least 50% sequence identity as a threshold cutoff. Sequence and structural superposition of the above entries confirmed that the Gly600 residue is conserved amongst all of them (Figure 8). The novelty herein lies with the insights that the 3D modelling provides on a structural level, and inevitably on the overall functioning level of the protein.

## 4. Discussion

AEC syndrome is a rather grave type of ectodermal dysplasia. Most patients display severe erosive lesions of skin at or after birth (>70% of patients), ankyloblepharon (70%), cleft lip and/or palate (80%), defects in nails and teeth, and other ectodermal defects including hypohidrosis, which increases the risk of hyperthermia and heat stroke [2]. They also suffer from hearing impairment, genitourinary deformities, and, rarely, from limb defects. Nevertheless, usually, AEC is a syndrome with a relatively good prognosis, granted that the symptoms can significantly improve over time.

The treatment of AEC depends on the particular clinical manifestations of each patient. Skin erosions with recurrent infections can be treated by a pediatric dermatologist. Scalp erosion is a manifestation of AEC with problematic treatment; treatment is fraught with failure due to the underlying pathology of the p63 mutation causing dysfunctional wound healing. Step-by-step approaches to treatment are frequently used, starting with daily baths, minor debridement, and emollients, working up to substantial skin excision. Skin grafting may be a part of the treatment, although it is very rarely performed because of a low success rate, and defective healing often leads to infections that necessitate rigorous debridement and antibiotic therapy [14]. Treatment with a p53-reactivating compound reportedly allowed cell differentiation and re-epithelialization of the eroded skin, resulting in loss of pain after few weeks [15]. Dental treatment utilizes prosthetic appliances initially attached to primary dentition and later to permanent premolars and/or molars [16,17]. In adolescence, evaluation of the permanent dentition and possible orthodontic therapy for expansion of the maxillary or mandible arch may be performed prior to implants and permanent prosthetic treatment [16].

The key to proper treatment and the associated good prognosis of AEC is an early diagnosis based on awareness of the clinical picture of the syndrome. Differential diagnosis should consider also EEC and Rapp–Hodgkin syndrome, both of which have many common features with AEC [18]. The presence of ankyloblepharon and erosive scalp lesions in most cases differentiates AEC from the other two syndromes, although some rare cases with overlapping clinical phenotypes have been reported [19,20]. In most instances, severe limb malformations, such as ectrodactyly, are present only in EEC patients. Interestingly, a case of EEC syndrome with an erosive presentation was reported in 1992, and postulated to represent a new syndrome [21]. Although the hypothesis of a new syndrome was later rejected, it is possible that this is the first case of EEC syndrome with scalp dermatitis [22]. All these syndromes are caused by dominant mutations in the *TP63* gene, which encodes a protein with five functional regions: the N-terminal transactivation domain, DNA-binding domain, oligomerization domain, sterile-α motif (SAM) domain, and C-terminal transactivation inhibitory (TI) domain [23,24,25]. EEC syndrome is caused by mutations in the DNA-binding domain, while AEC and RHS syndromes are caused by mutations in the SAM and TI regions [26,27,28].

The SAM domain of p63 may be implicated in protein–protein interactions with over 40 other proteins that have a crucial role in the control of transcription and ectodermal development [9,10,11]. Only those p63 isotypes with dominant negative properties contain the SAM domain. The unique phenotype of AEC syndrome seems to be a result of a disturbance of the connection between the SAM domain and other proteins, according to structural and functional research [11]. All known *TP63* gene mutations located in the SAM domain cause the skin abnormalities of AEC syndrome by abolishing interaction with ABBP1, and by modulating the FGFR-2 pathway, which leads to aberrant keratinocyte proliferation, differentiation and survival [11,12].

We report here a clinical and molecular genetic analysis of a typical case of AEC syndrome in a European (Italian/Greek) family. The studied case included a patient with the rare autosomal dominant AEC syndrome caused by a de novo mutation in the *TP63* gene. The detected point mutation c.1799G>T in exon 14 in the SAM domain of the gene caused an amino acid substitution G600V (p.Gly600Val). According to HGMD 2021.4, the c.1799G>T mutation (HGMD number CM0910988) was previously reported as G561V in a patient with AEC [13]. Therefore, the mutation c.1799G>T may be considered pathogenic, based on American College of Medical Genetics (ACMG) criteria for classifying pathogenic variants [29]. There are two pathogenic strong (PS) criteria, namely PS1 (same amino acid change as a previously established pathogenic variant) and PS3 (in vitro or in vivo studies supportive of a damaging effect on the gene product). A skin biopsy derived from a non-lesional region of the AEC patient with the same mutation revealed very reduced levels of PERP protein in the epidermis [13]. The *PERP* gene is a prime p63 target, and its protein plays a critical role in cell–cell adhesion and the integrity of stratified epithelia, due to its participation in complexes of adhesion desmosomes. While the uppermost layers of the patient’s skin remained stable, a huge diminution in PERP membrane staining in the basal and suprabasal layers was observed, suggesting that disrupted PERP expression may possibly cause some of the symptoms of AEC [13].

A point mutation c.1798G>C has been reported in an AEC case affecting the same codon by causing an amino acid substitution G600R (p.Gly600Arg) in the SAM domain [19]. In addition, there are other patients with AEC syndrome that also have amino acid substitutions similar to our patient (i.e., Glycine to Valine), in the same region of the SAM domain known to mediate protein–protein interactions [30]. Mutation G557V was detected in a patient with no published clinical data [31], and mutation G569V was found in a 7-year-old girl who presented with extensive erosive skin lesions from birth, a cleft lip and palate, ankyloblepharon, mid-face hypoplasia, and mild bilateral syndactyly of the third and fourth toes [9]. At the age of 4 years, other features became apparent in the girl, including hypodontia, corneal scarring, and conductive deafness [9]. Interestingly, an immunohistochemical analysis of this patient’s skin biopsy revealed some transactivation potential of mutant p63 [9]. As the mutation was predicted to be on the surface of the SAM domain, it likely compromises precise protein–protein interactions rather than causing large-scale structural perturbations of p63.

We performed a molecular mofiledeling analysis to possibly link the effect of the missense mutation G600V, detected in our patient, on the protein structure level. We noted that the introduction of the bulkier Valine residue in place of the slim Glycine residue caused a significantly altered 3D conformational arrangement of that protein region, pushing away the adjacent antiparallel α helix. We propose that the locally altered structure of the G600V mutant p63 introduced herein has a significant functional effect on specific protein–protein interactions, thus affecting the clinical phenotype. Previously observed phenotypic and immunostaining findings in a patient with similar Glycine to Valine substitution (G569V) in the same region of the SAM domain may corroborate this notion.

The modeled molecular and functional characterization of the G600V mutation of human p63 may pave the way for future investigations, and for the possible discovery of potential molecular modulators capable of reversing the introduced structural and functional deficit of this and other mutations in the SAM domain that affect protein–protein interactions. This knowledge may potentially present a clinical therapeutic window for Ankyloblepharon-ectodermal dysplasia-cleft lip/palate syndrome.

In our studied case, proper clinical examination and genetic counseling preceded genetic testing. After the detection of the responsible mutation, the parents learned, with relief, the accurate diagnosis, and their period of uncertainty ended. As a result, the parents considered the whole experience to be constructive; they viewed their daughter’s condition realistically, and realized that they should cooperate closely with the specialists who will contribute to the best possible management of their child.

## Data Availability

The original contributions presented in this study are included in the article. Further inquiries can be directed to the corresponding author.
